# Dampened virulence and limited proliferation of *Batrachochytrium salamandrivorans* during subclinical infection of the troglobiont olm (*Proteus anguinus*)

**DOI:** 10.1038/s41598-020-73800-y

**Published:** 2020-10-05

**Authors:** Zhimin Li, Elin Verbrugghe, Rok Konstanjšek, Maja Lukač, Frank Pasmans, Ivan Cizelj, An Martel

**Affiliations:** 1 Wildlife Health Ghent, Department of Pathology, Bacteriology and Avian Diseases, Faculty of Veterinary Medicine, Ghent University, Salisburylaan 133, 9820 Merelbeke, Belgium; 2 Department To Biology, Biotechnical Faculty, University of Ljubljana, Jamnikarjeva 101, 1000 Ljubljana, Slovenia; 3 Department of Poultry Diseases With Clinic, Faculty of Veterinary Medicine, University of Zagreb, Heinzelova 55, 10000 Zagreb, Croatia; 4 Zagreb Zoo, Maksimirski Perivoj Bb, 1000 Zagreb, Croatia

**Keywords:** Herpetology, Fungal pathogenesis

## Abstract

Emerging infections add to existing threats to the survival of amphibians worldwide. The olm (*Proteus anguinus*) is a vulnerable, troglobiont urodele species with a small European range and restricted to underground karstic systems. Population declines to emerging threats like the chytrid fungus *Batrachochytrium salamandrivorans*, are likely to go unnoticed due to inaccessibility of the species’ habitat. We here studied the interaction between olms and *B. salamandrivorans*. Experimental inoculation of olms resulted in low-level, asymptomatic but persistent infections, with limbs as predilection sites. The lack of exponential fungal growth in the olms’ epidermis correlated with limited fungal proliferation and dampened virulence gene expression after exposure to olm skin compounds. The olm is one of few western Palearctic urodeles that is tolerant to *B. salamandrivorans* infection and may act as a subterranean disease reservoir, yet costs of subclinical infection may compromise olm fitness on the long term.

## Introduction

During the past decades, around 500 amphibian species have declined in Australia, Europe and America due to the fungal disease chytridiomycosis^1^, which is caused by two chytrid fungi *Batrachochytrium dendrobatidis*^2^ and *Batrachochytrium salamandrivorans*^3^. Since first reported in 2010, *B. salamandrivorans* has brought multiple salamander populations to the edge of extinction^4^ and recently emerged in Spain, over 1000 km from the index outbreak site in the Netherlands^5^. This infection continues to pose a threat especially to small range susceptible salamanders^3,6^. Intrinsic host susceptibility is a major predictor of disease severity and impact, and current evidence suggests the level of susceptibility to be rather consistent within a given amphibian species^7^. Levels of susceptibility to *B. salamandrivorans* have been classified as: resistant (no infection, no clinical disease), tolerant (infection, no disease), susceptible (infection resulting in clinical disease, with the potential to recover) and lethal (death in the majority of infected animals)^7^.

Until the discovery of a cave fish^8^, the olm, *Proteus anguinus*, was the only known, true troglobiont vertebrate in Europe, which inhabits the underground water of Dinaric Karst in Italy, Slovenia, Croatia, Bosnia Herzegovina and Montenegro^9,10^. Olms have been listed as Vulnerable on the IUCN red list and list of EDGE of Existence program^11^ (https://www.edgeofexistence.org/species/olm/). Although it is protected by national legislation in its range states, the population is declining because of water pollution and habitat disturbance from land use changes occurring above the cave systems^12^. Proactive identification of threats to their survival is necessary since, due to their underground occurrence, olm populations are notoriously difficult to monitor. Therefore, we here estimated the threat of *B. salamandrivorans* to olms by a series of laboratory studies into the host–pathogen interaction.

## Results

### *Batrachochytrium salamandrivorans* binds to olm skin and subclinically persists in keratinized toes of olms

**Figure 1:**
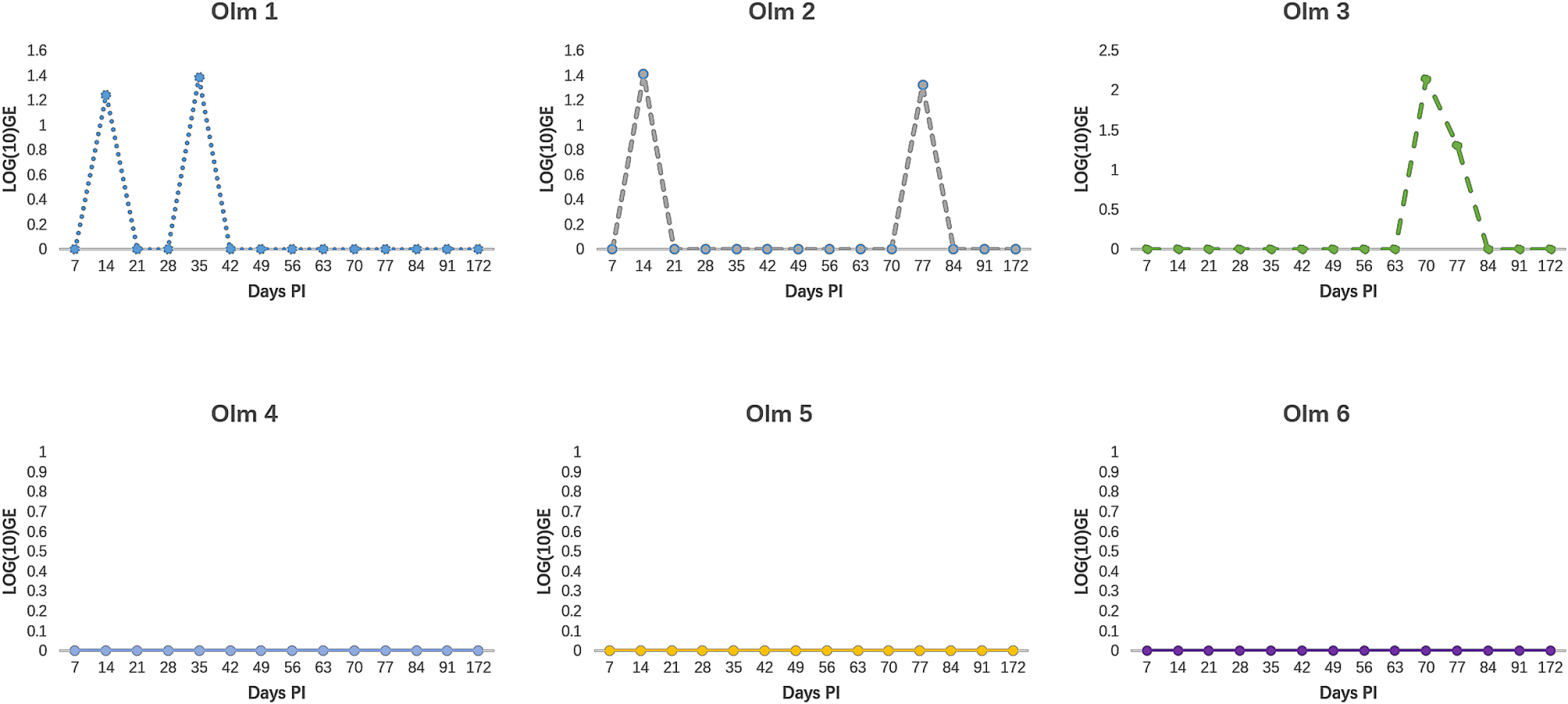
GE load dynamics in 6 olms after experimental exposure to *B. salamandrivorans*. Infection loads are shown as log_10_ GE values. Different individuals are shown in different graphs.

To assess the threat of *B. salamandrivorans* to olms, 6 animals were experimentally exposed to this fungal pathogen. None of the olms died during the 6 months after inoculation or developed any obvious clinical signs of infection. Using qPCR on skin swabs, only three of six animals were shown to develop low level infections, with intermittent fungal shedding (Fig. 1).

**Figure 2:**
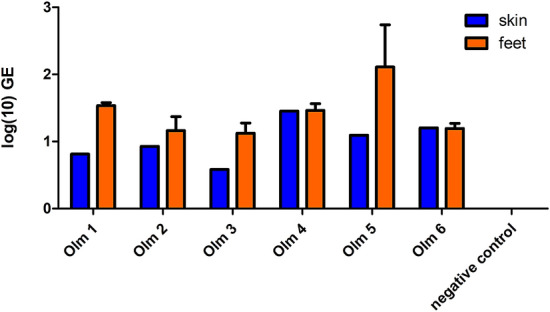
Log_10_ GE load in abdominal skin (n = 1) and feet samples (n = 3) of 6 experimentally infected olms and an uninfected animal (negative control).

After euthanasia, qPCR revealed that both feet and abdominal skin samples from all 6 animals were positive for *B. salamandrivorans* (Fig. 2).

**Figure 3:**
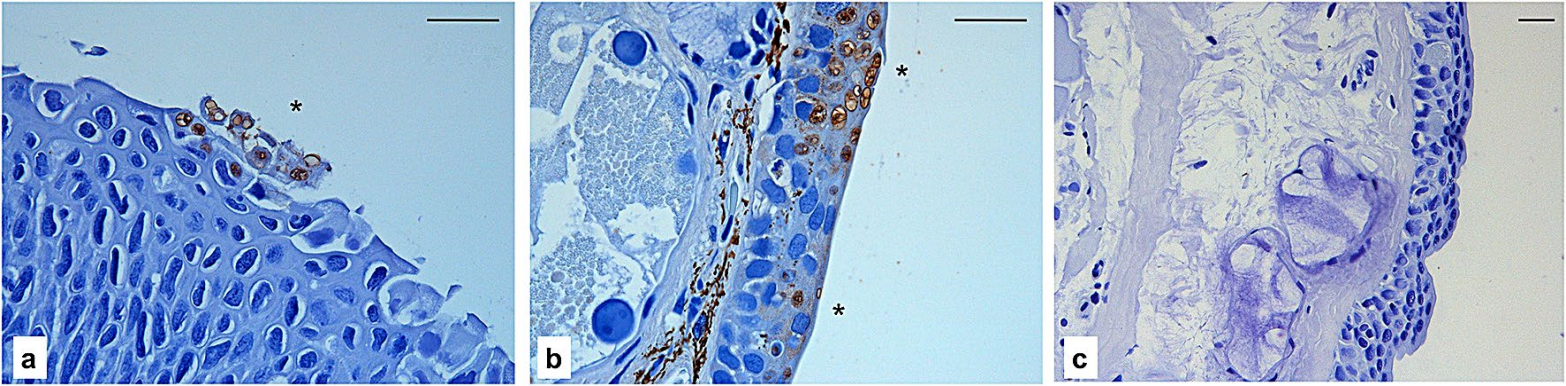
Immunohistochemistry of a toe of an olm (**a**) and the ventral skin of a fire salamander (**b**) that were experimentally infected with *B. salamandrivorans*. (**c**) shows the ventral skin of an uninfected olm. Brown staining shows the presence of intra-epidermal thalli of *B. salamandrivorans* (*) (scale bar, 50 μm).

The presence of *B. salamandrivorans* thalli in olm epidermis was confirmed using immunohistochemistry in the abdominal skin and in one foot of two animals, in the absence of any signs on histopathology (Fig. 3a). Thalli were located in the superficial epidermal layers, opposed to the pan-epidermal localization of fungal thalli in skin of affected fire salamanders (Fig. 3b). No thalli were observed in negative control (Fig. 3c).

### *Batrachochytrium salamandrivorans* attaches to olm skin but the skin mucosome limits fungal proliferation

**Figure 4:**
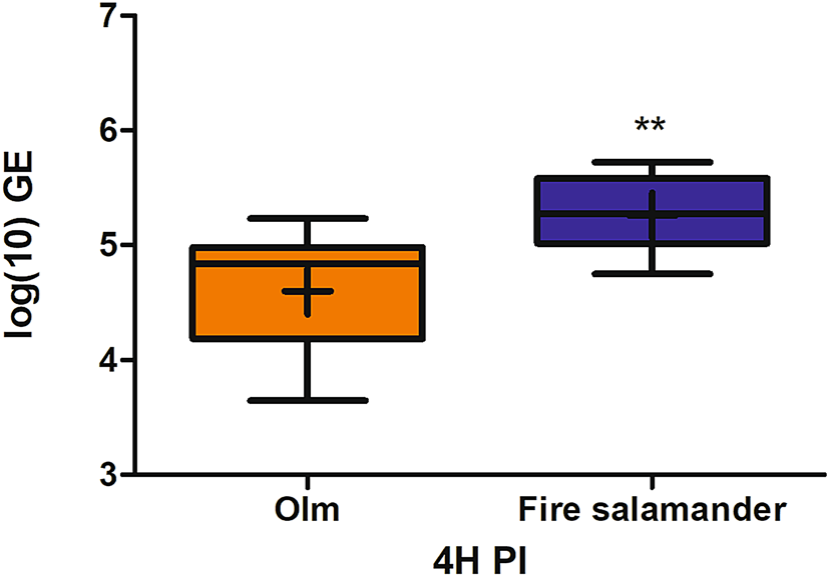
GE loads of *B. salamandrivorans* attached to skin biopsies of fire salamanders (n = 18) and olms (n = 18) after co-incubation for 4 h. The whiskers represent the median, the minimum and maximum values, and the first and third quartiles. A cross (+) indicates the mean value. **P < 0.001.

**Figure 5:**
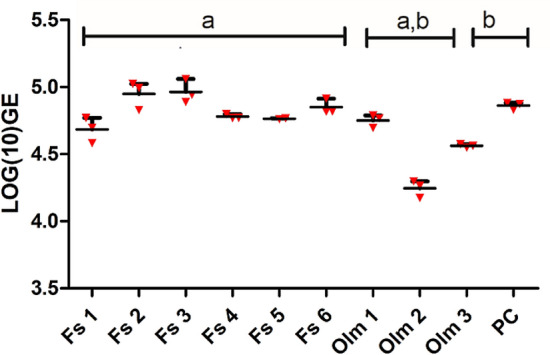
Growth of *B. salamandrivorans* in mucosomes collected from olm and fire salamander. qPCR results are shown in Log_10_ GE value. The whiskers represent the mean with range of 3 replicates. Same letter shared in groups indicates significant difference (*P* < 0.05) between groups. *Fs* fire salamander, *PC* positive control.

To examine the role of the mucosome as a first line of defense against *B. salamandrivorans*, growth control capacities of both fire salamander and olm mucosome were evaluated. In the invasion trial with skin tissue, *B. salamandrivorans* spores attached to both olm and fire salamander skin in 4 h. The GE loads in skin samples from fire salamanders were significantly (*P* = 0.0023) higher than those in skin samples from olms (Fig. 4). In contrast to the fire salamander mucosome, the olm mucosome showed significant inhibition of the growth of *B. salamandrivorans* compared to the positive control (Fig. 5).

### Dampened virulence of *B. salamandrivorans* after exposure to olm skin

**Figure 6:**
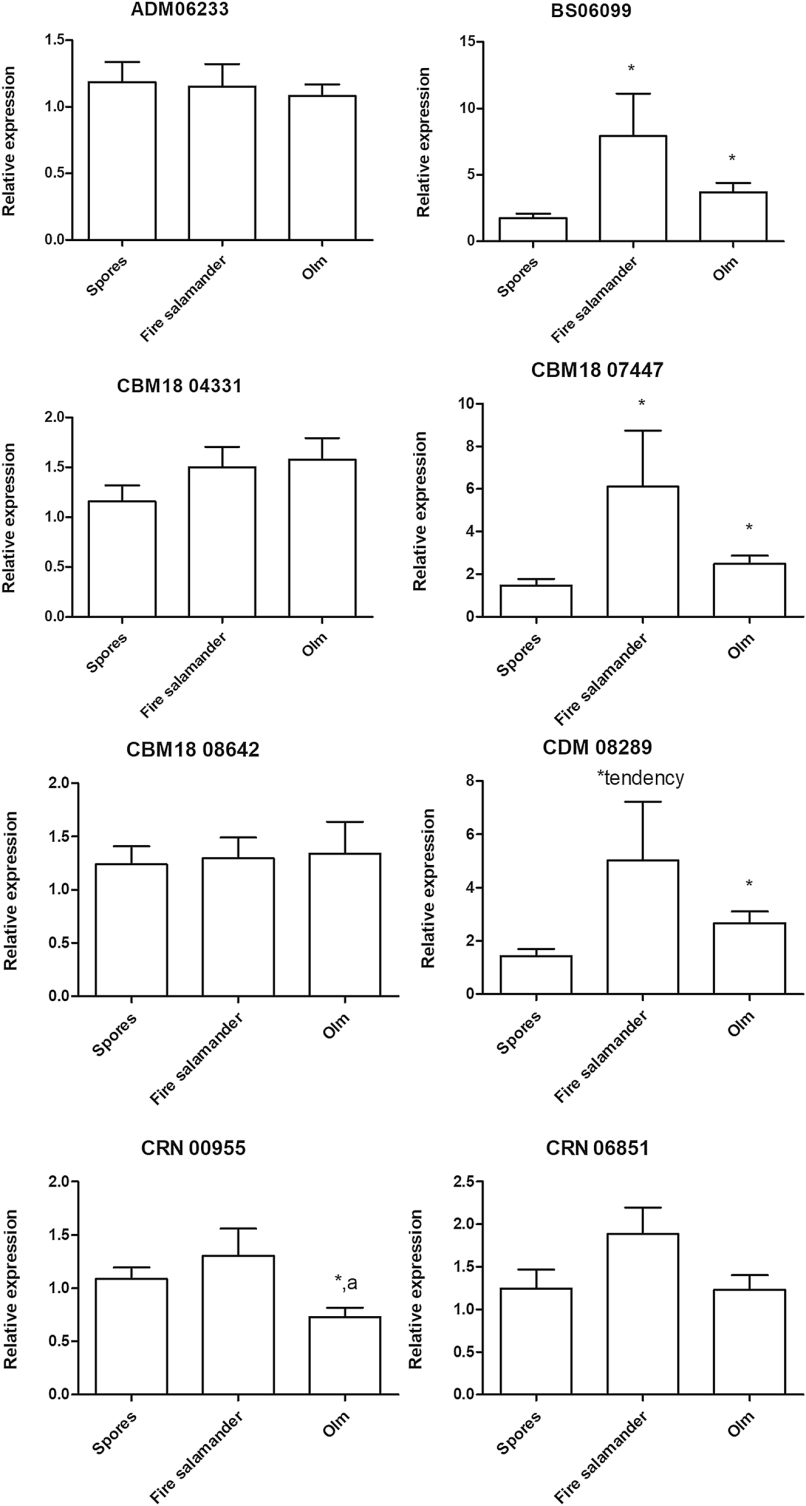
Relative expression profiles of *B. salamandrivorans* virulence genes after incubation of zoospores with fire salamander skin (n = 18) or olm skin (n = 18) for 2 h, compared to gene expression of zoospores that were not exposed to salamander skin (“spores”). The results are presented as means + standard error of the mean (SEM), asterisk shows the significance (*P* < 0.017) compared to the control group (“spores”), the letter “a” shows a significant (*P* < 0.017) difference between olm and fire salamander.

Searching for changes in *B. salamandrivorans* pathogenicity, we examined the expression of a selection of *B. salamandrivorans* virulence genes in spores and spores that were first exposed to olm or fire salamander skin. Compared to control spores that were not exposed to host tissue, Bs 06099, CBM 18 07447 and CDM 08289 were (tended) significantly upregulated in *B. salamandrivorans* spores after exposure to both the fire salamander (*P* = 0.003, *P* = 0.017, *P* = 0.026, respectively) and the olm skin (*P* = 0.006, *P* = 0.19, *P* = 0.004, respectively) (Fig. 6). While CRN 00955 from the Crinkler family showed no altered expression in fire salamanders, it was significantly (*P* = 0.016) downregulated in olm. For the other genes, CRN 06851, ADM 06233, CBM 18 04331 and CBM 18 08642, there was no significant difference between different groups.

## Discussion

In contrast to the vast majority of western Palearctic urodele species assessed, olms were shown to be tolerant to persistent *B. salamandrivorans* infection after experimental exposure and none of the infected animals developed any obvious signs of disease. Our results suggest olms to be potential long-term but low-level carriers of *B. salamandrivorans*. Although extrapolation to natural conditions should be done with care, the constant environment in cave systems, similar to our experimental conditions, and supposedly increased stress in the captive animals render a scenario of severe disease and mortality under natural conditions unlikely. With the current information, we therefore estimate the risk of *B. salamandrivorans*-driven declines in olms as minor. However, the long term proliferation of the fungus within the olm keratinized limb tissue may coincide with a more subtle cost, associated with increased energy expenditure, impaired locomotion or increased vulnerability of the limbs to secondary infection.

These low level but persistent infections that are limited to the most superficial epidermal layers without causing the skin erosions observed in susceptible hosts^3^ correlate with results obtained in the in vitro experiments. The olm mucosome was shown to limit fungal proliferation. The amphibian mucosome is made up of host skin compounds and symbiotic bacterial products^13^ and has been suggested to play a role as first line of defense against *B. salamandrivorans*^14^. Besides limiting fungal proliferation, skin compounds may play an additional role by trapping of spores in the mucus layer with subsequent removal due to the constant shedding of skin mucus. This mechanism is well known to be an important defense mechanism in the vertebrate intestinal tract^15^.

Besides limiting fungal growth, contact of fungal spores with olm skin dampened fungal virulence. Virulence gene expression was different between (tolerant) olms and (lethally susceptible) fire salamanders. The most obvious difference was noticed in Crinkler gene CNR 0095 expression. This gene was downregulated after contact of the spores with olm skin. These results suggest that pathogen virulence gene expression in the tolerant olms is regulated such that fungal populations are kept within limits, without obvious damage to the host as observed in the infection trial. Further elucidation of the role of the presumed virulence genes in *B. salamandrivorans* chytridiomycosis is likely to produce key insights in the host–pathogen interaction.

## Materials and methods

All methods were carried out in accordance with relevant guidelines and regulations.

### Infection dynamics of *B. salamandrivorans* in experimentally infected olms (*Proteus anguinus*)

In a first experiment, we wanted to quantify infection and disease dynamics of *B. salamandrivorans* infections in six olms. The olms originated from Slovenia (3 animals) and Croatia (3 animals), from captive colonies that consisted of animals that were flushed outside their cave habitat. The animals were housed individually in aquaria at 11 °C in complete darkness and were left to acclimate for 6 weeks. Aquaria contained 3 L of aged tap water, which was replaced weekly, and a hiding place. Animals were fed once weekly with tubifex. All animals were clinically healthy and negative for *B. salamandrivorans*, *B. dendrobatidis* and Ranavirus (assessed using qPCR on skin swabs following standard protocols^16–18^) at the start of the experiment. Experimental infection consisted of exposure to a single dose of 1.5 × 10^3^ zoospores of the *B. salamandrivorans* type strain (AMFP13/1) in 620 mL water for 24 h^3^. After exposure, animals were inspected daily for clinical signs and sampled weekly to monitor *B. salamandrivorans* infection dynamics using qPCR on skin swabs^18^. The experiment was ended at 6 months after inoculation. All animals were euthanised and examined for the presence of *B. salamandrivorans* infection using qPCR on skin tissue samples (abdominal skin (1 sample per animal) and feet (3 samples per animal)) and immunohistochemistry (abdominal skin and feet)^18,19^. Skin tissue of an uninfected olm was included as a negative control for qPCR and immunohistochemistry analysis. To confirm the specificity of the qPCR reactions, we randomly selected 4 positive qPCR products for sequencing^18^. The infection experiment was approved by the ethical committee of the Faculty of Veterinary Medicine (Ghent University) (EC2017/75).

### Olm mucosome activity against *B. salamandrivorans*

By skin mucosome, we refer to the compounds present at the surface of the amphibian skin, which can be rinsed of with water. Mucosomes were collected from healthy and *B. salamandrivorans* naïve olms (3) and fire salamanders (*Salamandra salamandra*) (6) using the bathing method described by Woodhams^20^. Briefly, each animal was bathed in a petri dish with HPLC water for 1 h, the volume of water was calculated according to animal surface^21^. After filtration through a 0.2 μm pore filter (Whatman, GE Healthcare Life Sciences), the collected mucosomes were kept on ice. Meanwhile, *B. salamandrivorans* spores were collected from culture flasks with sporulating sporangia by flooding the flask with TGhL medium. The collected spores were filtered using a sterile filter with pore size 10 μm (PluriSelect, Leipzig, DE). To achieve the target concentration of 1 × 10^6^ spores per mL, the spore suspension was diluted with TGhL before use. Finally, 100 µL of this spore suspension was used to inoculate 10^5^ spores per well of a 96-well flat-bottom plate (Greiner BIO-ONE, Stonehouse, Gloucestershire, UK).

In each well, 100 μL of the mucosome solution was added to the spore suspension. In positive control wells, filtered HPLC water was added instead of mucosome. All the conditions were performed in triplicate. The plate was incubated at 15 °C. After 5 days of incubation, the bottom of each well was scraped with a 100 μL tip and the liquid was transferred to a 1.5 mL Eppendorf tube for DNA extraction and qPCR^18^. Multiple comparisons on the original values were assessed by a Kruskal–Wallis analysis, followed by pairwise Mann–Whitney U-test (SPSS version 25; SPSS Inc., Chicago, IL, USA).

### Skin attachment and virulence gene expression of *B. salamandrivorans*

We here assessed the regulation of virulence mechanisms of *B. salamandrivorans* and its ability to attach and invade the skin of olms versus fire salamanders as a proxy for pathogenicity and colonization capacity^7^ using an ex vivo protocol modified from Van Rooij et al.^22^. Three 5 mm and three 8 mm diameter, full thickness ventral skin biopsies were collected from each of the six olms of the infection trial after euthanasia, and from six *B. salamandrivorans* negative fire salamanders.

*Batrachochytrium salamandrivorans* zoospores were collected from mature cultures in sterile distilled water. The water containing zoospores was filtered using a sterile filter with pore size 10 μm. To achieve the target concentration of 2.5 × 10^7^ spores per mL, the spore suspension was concentrated after centrifugation at 3000 g for 5 min at 15 °C.

To quantify *B. salamandrivorans* invasion in host tissue, the 8 mm biopsies were transferred to wells of a 96 well plate, exposed to 200 μL spore suspension and incubated for 4 h at 15 °C. Then, the tissues were rinsed with sterile distilled water once to get rid of unattached spores and cut into two equal pieces. One piece was transferred to an empty Eppendorf to analyse with *B. salamandrivorans* qPCR^18^, the second piece was processed for immunohistochemical staining^19^. DNA was extracted with a DNeasy Blood & Tissue Kit (Qiagen, Germantown, USA) and qPCR was performed as described by Boyle et al.^16^ and Blooi et al.^18^. To compare association of *B. salamandrivorans* between hosts, the original log_10_ genomic equivalent (GE) values were assessed using a Kruskal–Wallis analysis (SPSS version 25).

To quantify virulence gene expression of *B. salamandrivorans* after exposure to host skin tissue, the 5 mm skin biopsies were transferred to individual 2 mL Eppendorf tubes, exposed to 300 μL of the zoospore suspension and incubated at 15 °C for 2 h. We analyzed gene expression of a selection of virulence genes as identified by Farrer et al.^24^. Gene expression after exposure to host tissue was compared to gene expression of zoospores without tissue exposure. Total RNA extraction was performed using the Qiagen RNeasy mini Kit following the standard protocol^23^. The concentration and quality of the RNA was checked with the Agilent 2100 Bioanalyzer System (Agilent Technologies, Waldbronn, Germany). RNA (500 ng) was reverse transcribed to cDNA with the iScript cDNA synthesis kit (Bio-Rad, Hercules, CA) and cDNA was stored at − 20 °C before use. Real-time quantitative PCR reactions were run in duplicate and the reactions were performed in 10 μL volumes using the iQ SYBR Green supermix (Bio-Rad) and 1.5 μL 1/5 diluted cDNA. The experimental protocol for qPCR was performed on a CFX384 RT-qPCR System with a C1000 Thermal Cycler (Bio-Rad). The results were analyzed using the Bio-Rad CFX manager 3.1. Quantification cycle (Cq) values were obtained using auto baseline settings and they were applied per primer set. The threshold for maximum Cq difference between the technical replicates was set to 1.

The stability of candidate reference genes (*GAPDH*, *TUB*, *a-centractin*)^23^ was analyzed using QBase, all showing a GeNorm M value ≤ 1.0 and a coefficient of variation value ≤ 0.5. Relative gene expression analysis of the target genes^24^ is shown as fold changes of mRNA expression, which were calculated based on the CNRQ values obtained in QBase. Since the data were not normally distributed, a non-parametric Kruskal–Wallis analysis on the CNRQ values was performed, followed by a pairwise Mann–Whitney U-test (SPSS version 26), with a Bonferroni-corrected *P* value (*P* value = 0.05/number of comparisons). The *P* value for significance was set at 0.05/3 = 0.017, and for tendency at 0.1/3 = 0.033.

## Data Availability

The datasets generated during and/or analysed during the current study are available from the corresponding author on reasonable request.
